# Dr. Salina P. Colegrove

**Published:** 1918-12

**Authors:** 


**Dr. Salina P. Colegrove,** Buffalo 1888, died at her home in Salamanca, Oct. 21.

